# Influence of Obesity on Blood Pressure in Young Individuals: A Cross-Sectional Study

**DOI:** 10.7759/cureus.91570

**Published:** 2025-09-03

**Authors:** Sujatha Talikoti, Anurag Rawat, Girish Manohar Chavan, Rainita Pise, Amrit Podder, Jayballabh Kumar, Sreemoyee Dutta, Sonali Dash, Sariya Nazim

**Affiliations:** 1 Department of Physiology, Naraina Medical College and Research Centre, Kanpur, IND; 2 Department of Cardiology, Himalayan Institute of Medical Sciences, Dehradun, IND; 3 Department of Community Medicine, Bharati Vidyapeeth (Deemed to Be University) Medical College and Hospital, Sangli, IND; 4 Department of Community Medicine, Datta Meghe Medical College, Nagpur, IND; 5 Department of Physiology, Teerthanker Mahaveer Medical College and Research Centre, Moradabad, IND; 6 Department of Peridontology, Kalinga Institute of Dental Sciences, Bhubaneswar, IND; 7 Department of Cardiology, Rajiv Gandhi Super Speciality Hospital (RGSSH), Delhi, IND

**Keywords:** blood pressure, body mass index, cardiovascular risk, hypertension, obesity

## Abstract

Introduction: Obesity has emerged as a significant public health issue worldwide, with increasing evidence pointing toward its association with elevated blood pressure and early onset of cardiovascular disease. In India, the prevalence of obesity among the young adult population is rising, necessitating timely research into its impact on hemodynamic parameters. Our study aims to explore the relationship between obesity and blood pressure phenotypes among young individuals aged 18 to 25 years using body mass index (BMI) as a measure of adiposity.

Materials and methods: A cross-sectional study was conducted among 150 young males aged between 18 and 25 years in a medical college in India. Participants were divided into three equal groups based on BMI: Group 1 (normal BMI), Group 2 (overweight), and Group 3 (obese), with 50 participants in each group. Systolic blood pressure (SBP), diastolic blood pressure (DBP), pulse pressure (PP), and mean arterial pressure (MAP) were measured. Data analysis was performed using IBM SPSS Statistics for Windows, Version 27 (Released 2020; IBM Corp., Armonk, New York). ANOVA was used to compare blood pressure phenotypes across groups, and Spearman's correlation was used to assess the relationship between BMI and blood pressure parameters.

Results: A statistically significant increase in all blood pressure phenotypes (SBP, DBP, PP, MAP) was observed with increasing BMI (p < 0.001). Group 2 participants had higher blood pressure than Group 1, while Group 3 participants showed significantly elevated values compared to both Group 1 and Group 2. A positive correlation was identified between BMI and SBP (r = 0.61), DBP (r = 0.58), PP (r = 0.49), and MAP (r = 0.62), all with p-values < 0.001.

Conclusion: Obesity in young adults is significantly associated with elevated blood pressure. Early identification and intervention may prevent the onset of hypertension and its complications in later life.

## Introduction

The global obesity epidemic is one of the most pressing health concerns of the 21st century. According to the World Health Organization (WHO), obesity has nearly tripled since 1975, and its prevalence continues to grow among children and young adults alike [1]. In India, the rising incidence of overweight and obesity, particularly among young people, is contributing to an earlier onset of lifestyle diseases, including hypertension, diabetes, and cardiovascular disorders [2]. Hypertension, once considered a disease of older adults, is increasingly being diagnosed in younger individuals. Elevated blood pressure at a young age has been associated with adverse cardiovascular events and increased all-cause mortality later in life [3]. The pathophysiology linking obesity to hypertension includes mechanisms such as sympathetic nervous system activation, renin-angiotensin-aldosterone system (RAAS) dysregulation, endothelial dysfunction, and inflammation [4]. Body mass index (BMI) is a commonly used indicator for assessing obesity. Several studies have reported a positive correlation between BMI and blood pressure [5-7]. However, limited data are available on this association among the Indian young adult population. This study was undertaken to bridge this knowledge gap and evaluate the influence of obesity, categorized by BMI, on blood pressure parameters among individuals aged 18 to 25 years.

## Materials and methods

Study design and setting

This was a cross-sectional observational study [8] conducted in the Yoga Lab of the Department of Physiology, Teerthanker Mahaveer Medical College & Research Centre, Teerthanker Mahaveer University, Moradabad, Uttar Pradesh, India, between January and June 2025.

Study population and sampling

A total of 150 young males aged 18-25 years were recruited through convenience sampling. All participants were enrolled and categorized based on BMI into three equal groups. Group 1 consisted of participants of normal weight with a BMI of 18.5-24.9 kg/m², group 2 participants were overweight with a BMI of 25.0-29.9 kg/m², and group 3 consisted of obese individuals with a BMI ≥30.0 kg/m². Each group comprised 50 participants [9].

Ethical considerations

The study was conducted after obtaining approval from the Institutional Ethics Committee of Teerthanker Mahaveer University (Ref No: TMU/IEC/2024-25/FACULTY/23, dated 01/12/2024). All participants were enrolled in the study after providing voluntary informed written consent.

Inclusion and exclusion criteria

All male participants aged between 18 and 25 years who had no known history of hypertension, cardiovascular disease, diabetes, or chronic illness, and were not on any medication affecting blood pressure, were included in the study. Individuals with endocrine disorders, renal disease, or known secondary causes of hypertension were excluded. We also excluded individuals with a history of smoking or alcohol intake.

Measurement of the study variables

Height (cm) and weight (kg) were measured using a standard stadiometer and weighing scale, respectively, and BMI was calculated. Systolic blood pressure (SBP) and diastolic blood pressure (DBP) were recorded in the right arm in a sitting position after a 10-minute rest using a calibrated mercury sphygmomanometer. Three readings were taken at 10-minute intervals, and the average of the readings was recorded. Pulse pressure (PP) and mean arterial pressure (MAP) were derived from SBP and DBP values [9].

Statistical analysis

The data were stored in a Microsoft Excel spreadsheet and analyzed using IBM SPSS Statistics for Windows, Version 27 (Released 2020; IBM Corp., Armonk, New York). Descriptive statistics (mean ± SD) were calculated. ANOVA was employed to compare the means of BP parameters across the three study groups. Post hoc analysis was applied for inter-group comparisons. Spearman’s correlation test was used to assess the relationship between BMI and blood pressure variables. A p-value <0.05 was considered statistically significant.

## Results

The baseline characteristics of all 150 participants (n = 50 in each group) are presented in Table 1, which shows that there was no significant difference (p > 0.05) in the age of participants between the study groups. The BMI of group 2 was higher than that of group 1, whereas the BMI of group 3 was higher than that of group 2 and group 1. From the observed values in Table 1, it is evident that the proposed study design was properly followed.

The blood pressure variables of all participants across groups are shown in Table 2. It is apparent from the table that the mean values of all blood pressure variables in group 2 were significantly (p < 0.05) higher than those in group 1, whereas the values in group 3 were significantly higher (p < 0.05) than those in both group 1 and group 2.

Table 3 represents the analyzed report of Spearman’s correlation between blood pressure variables and BMI. Our results show that BMI shares a positive correlation with all blood pressure variables. All correlations were statistically significant (p < 0.05).

**Table 1:** Baseline characteristics of study participants p < 0.05 was considered statistically significant. BMI, body mass index.

Variable	Group 1 (n = 50), mean ± SD	Group 2 (n = 50), mean ± SD	Group 3 (n = 50), mean ± SD	p-value
Mean age (years)	20.5 ± 1.4	20.6 ± 1.2	20.4 ± 1.3	>0.05
Mean BMI (kg/m²)	22.4 ± 1.3	27.1 ± 1.5	32.8 ± 1.7	<0.05

**Table 2:** Blood pressure variables across groups ANOVA showed statistically significant differences across all parameters. Post-hoc analysis confirmed that all intergroup differences were significant (p < 0.01). SBP, systolic blood pressure; DBP, diastolic blood pressure; PP, pulse pressure; MAP, mean arterial pressure.

Variable	Group 1 (n = 50), mean ± SD	Group 2 (n = 50), mean ± SD	Group 3 (n = 50), mean ± SD	p-value
SBP (mmHg)	112.2 ± 6.1	121.4 ± 5.9	130.5 ± 7.3	<0.001
DBP (mmHg)	72.6 ± 4.3	78.8 ± 4.6	85.3 ± 5.1	<0.001
PP (mmHg)	39.6 ± 3.9	42.6 ± 3.6	45.2 ± 3.8	<0.001
MAP (mmHg)	85.9 ± 4.2	93.0 ± 4.1	100.3 ± 4.6	<0.001

**Table 3:** Correlation between BMI and blood pressure phenotypes SBP, systolic blood pressure; DBP, diastolic blood pressure; PP, pulse pressure; MAP, mean arterial pressure; r,  Correlation coefficient; Spearman’s correlation was performed.

Variable (n = 150)	BMI (kg/m²)	p-value
SBP (mmHg)	r = 0.61	<0.001
DBP (mmHg)	r = 0.58	<0.001
PP (mmHg)	r = 0.49	<0.001
MAP (mmHg)	r = 0.62	<0.001

## Discussion

Our study demonstrates a statistically significant positive association between BMI and blood pressure among young adults. Participants classified as overweight and obese had higher SBP, DBP, PP, and MAP compared to those with normal BMI. These findings are consistent with prior studies that reported similar patterns of elevated blood pressure in obese individuals [5,6].

Mechanistic pathways for the observed results

The mechanisms underlying this relationship are multifactorial. Obesity is known to activate the sympathetic nervous system, increase leptin resistance, elevate circulating insulin levels, and induce RAAS activity, all of which contribute to increased vascular resistance and volume overload. Moreover, increased adipose tissue may produce inflammatory cytokines that exacerbate vascular stiffness and endothelial dysfunction [10].

This study underscores the need for early intervention strategies such as dietary modification, increased physical activity, and routine screening for blood pressure in young adults with high BMI [11,12]. Given the early onset of elevated BP in these individuals, failure to address modifiable risk factors could lead to premature cardiovascular morbidity and mortality [13,14].

Comparison with the existing literature

Our study corroborates similar findings from previous studies in this area. Zhang et al., in their study of 4135 college students, highlighted the importance of maintaining an optimal BMI for the prevention of hypertension [15]. Podder A et al. observed that obesity parameters are positively correlated with blood pressure and arterial stiffness [9].

Limitations of the study

Our study is cross-sectional in nature, which limits causal inference, and it also has a relatively small sample size. In addition, the study was conducted in a single center, which may limit the generalizability of the findings to different geographical areas. These are important limitations of the study.

Future directions

Although our study used BMI as a surrogate marker of adiposity, future studies should incorporate additional indices such as waist circumference, waist-hip ratio, and body fat percentage for more accurate risk stratification. Additionally, a larger multicentric longitudinal study could provide deeper insight into the causality and long-term cardiovascular risks associated with obesity in youth.

## Conclusions

This cross-sectional study provides strong evidence that obesity is significantly associated with increased blood pressure among young adults aged 18-25 years. The positive correlation between BMI and all blood pressure phenotypes highlights the urgent need for early screening, lifestyle interventions, and health education to mitigate long-term cardiovascular risks in this vulnerable age group. An interventional study with a larger sample size involving lifestyle modifications may offer further insight in this direction.
